# Gaucher Disease—Correlation of Lyso-Gb1 with Haematology and Biochemical Parameters

**DOI:** 10.3390/metabo15110731

**Published:** 2025-11-07

**Authors:** Simona D’Amore, Sneha Patel, Juniebel Cooke, Uma Ramaswami

**Affiliations:** 1 Lysosomal Storage Disorders Unit, Royal Free Hospital NHS Foundation Trust London, London NW3 2QG, UK; 2 Genetics and Genomic Medicine, University College London, London WC1N 1EH, UK

**Keywords:** Gaucher disease, lyso-Gb1, biomarker, enzyme replacement therapy, substrate reduction therapy

## Abstract

**Background/Objectives**: Gaucher disease (GD) is a lysosomal disorder caused by a deficiency of β-glucosidase. Disease-modifying therapies (DMTs) include enzyme replacement therapy (ERT) and substrate reduction therapy (SRT). Glucosylsphingosine (lyso-Gb1) is a biomarker with high sensitivity and specificity in GD. **Methods**: In GD patients attending a specialist centre, we evaluated dried blood spot lyso-Gb1 levels (normal values ≤ 6.8 ng/mL) by treatment status, sex, GD type and genotype, ERT dose, DMT type and duration, spleen status, and association with other GD biomarkers. **Results**: A total of 111 patients were screened; 100 (54M:46F; 93 GD1 and 7 GD3; median age 45.2 years, IQR 34.2–57.2; 7 naive and 93 patients on DMTs for a median of 10.4 years, IQR 5.7–21.2) had at least one lyso-Gb1 measurement. Median lyso-Gb1 values were higher in naïve (195, IQR 48.6–388) patients than treated patients (47.1, IQR 23.1–89.7), *p* = 0.015; higher in those treated ≥ 15 years (62.9, IQR 36.6–103) than in those treated < 15 years (35.1, IQR 20.3–73.9), *p* = 0.006; and higher in splenectomised (83.4, IQR 34.7–224.5) patients than non-splenectomised patients (40.7, IQR 21.4–77.1), *p* = 0.044. ERT dose > 60 U/kg had high median lyso-Gb1 values (87.3, IQR 19.7–126), reflecting greater disease burden, and this high dose was only used in patients with GD3. Lyso-Gb1 correlated with chitotriosidase (r = 0.495; *p* < 0.001) and haemoglobin (r = −0.231; *p* = 0.022). In a subset of 50 patients with paired values, lyso-Gb1 decreased from baseline (median −1.7 ng/mL, IQR −24.5–14.8). **Conclusions**: Whilst there was a modest decrease in lyso-Gb1 over time on DMTs, the values remained significantly above the normal range, which may be driven by underlying mechanisms such as inflammation.

## 1. Introduction

Gaucher disease (GD) is an autosomal recessive lysosomal disorder caused by biallelic mutations in the GBA1 gene, leading to a deficiency in the β-glucosidase enzyme [1,2,3]. As a result, glucosylceramide (GL1) and its deacylated substrate glucosylsphingosine (lyso-Gb1) accumulate in cells of the monocytes/macrophage lineage of various organs, such as spleen, liver, and bone marrow, leading to progressive hepatosplenomegaly, anaemia, thrombocytopenia, and skeletal disease [1,2,4,5,6]. Classically, GD is classified into three main phenotypes: the non-neuronopathic type 1 (GD1) with predominant haematologic, visceral, and bone involvement; the acute neuronopathic type 2 (GD2); and chronic neuronopathic type 3 (GD3) with primary central nervous system involvement, with GD2 being the most severe and usually fatal in infancy and GD3 being a later-onset and involving progressive neurological deterioration [1].

GD diagnosis relies on biochemical (demonstration of a deficient β-glucosidase enzyme activity in cells) and genetic (identification of pathogenic variants in GBA1) testing [1]. After the diagnosis of GD is made, a decision on when to start treatment should be made based on the patient’s genotype and the individual’s disease severity, symptoms, and progression, aiming to prevent the development of irreversible complications [7].

GD requires a multidisciplinary approach, which includes disease-modifying therapies (DMTs) such as enzyme replacement therapy (ERT) and substrate reduction therapy (SRT), and supportive care. ERTs have been proven effective in improving hepatomegaly and splenomegaly in both non-neuronopathic and chronic neuronopathic types; however, because ERT does not cross the blood–brain barrier, it cannot prevent neurological decline in GD3 [8,9]. SRTs include miglustat and eliglustat: eliglustat has demonstrated an efficacy similar to ERT [10,11,12,13,14], whilst miglustat use is limited by the occurrence of adverse effects such as diarrhoea, tremor, and peripheral neuropathy [15,16]. Therapeutic include those related to organ function, such as improving haemoglobin and platelet levels, reducing spleen and liver volume, preventing episodes of osteonecrosis and bone crises, resulting in improved patients quality of life [7].

Several biomarkers have been used for monitoring GD, such as ferritin, tartrate-resistant acid phosphatase, angiotensin-converting enzyme, chitotriosidase, and chemokine C–C motif ligand (CCL18), which, however, are not specific to GD [17,18,19,20]. Also, about 10% of the population a have the CHIT1 gene variant that results in deficient chitotriosidase [21,22]. Over the past few years, lyso-Gb1 has been shown to be a highly sensitive and specific biomarker for diagnosis and monitoring of treatment response in GD and has been proven to be superior to chitotriosidase and CCL18 [23,24,25]. Lyso-Gb1 has also been found to correlate with disease severity, with higher concentrations in patients with the L444P genetic variant, which is associated with a more severe disease, compared with subjects with the N370S variant, which is found in patients with a milder form of the disease [24].

The Lysosomal Storage Disorders Unit (LSDU) at the Royal Free London NHS Trust is a specialist centre in England that provides a one-stop service for the diagnosis and management of patients with an LSD, including GD. In GD patients attending the LSDU at the Royal Free London, we retrospectively evaluated dried blood spot (DBS) lyso-Gb1 levels (normal values < 10 ng/mL) by treatment status (naive/treated) and association with other GD biomarkers (i.e., haemoglobin levels, platelet count, ferritin, and chitotriosidase). Further analyses by sex, GD type, DMT type, ERT dose, and treatment duration were performed in the treated group.

## 2. Materials and Methods

### 2.1. Patients

Data for patients aged  ≥  18 years who had at least one lyso-Gb1 value and contemporaneous biochemistry were included in the analysis. Patients were stratified by treatment status (naïve, treated) relative to the time of the first lyso-Gb1 measurement (defined as the baseline). Naïve patients were those who had received no DMTs prior to the first lyso-Gb1 measurement, while the treated patients were those who had received either ERT or SRT and had not stopped treatment before the first lyso-Gb1 measurement. The treated cohort was further analysed by sex, GD type, DMT type (ERT; SRT), ERT dose (as units/kilogram) (<30 U/kg; 30–60 U/kg; >60 U/kg), treatment duration (<15 years; ≥15 years), and spleen status (with spleen; splenectomised). Biochemistry included full blood count and other biomarkers of GD activity (ferritin [normal values: 24–340 µg/L for males and 11–310 µg/L for females] and chitotriosidase [normal values: 0–150 nmol/hr/mL]). The data included in this manuscript focused on haematology, biochemistry, and organ involvement [26].

Clinical information from patients included in the study was extracted from medical records and entered into a protected database. See Table 1 for details.

### 2.2. Assessments

The blood samples were obtained from GD patients undergoing their follow-up visits. Lyso-Gb1 concentrations (normal values: ≤6.8 ng/mL) were analysed by Centogene DBS Assay (Rostock, Germany) using mass spectrometry of DBS samples as previously described [27].

### 2.3. Data Collection and Ethical Considerations

The retrospective data presented are from routine standard-of-care investigations and were obtained from electronic medical records and the LSDU database, where lyso-Gb1 data are recorded. As this is a service and standard-of-care evaluation study, it did not require ethics approval. Only clinicians who have authorised access to the data collated the information.

### 2.4. Statistical Analysis

Descriptive statistical analyses were used for demographic, treatment status (naive; treated), and treatment duration (<15 years; ≥15 years) across all patients. Categorical variables were expressed as proportions (%), continuous variables as medians with IQR. Comparison between groups was assessed with a *t*-test (two groups) or the Kruskal–Wallis one-way analysis of variance (more than two groups), followed by the Kruskal–Wallis multiple comparison z-value test (Dunn’s test) and with Pearson’s chi-squared test. The correlation between continuous variables was assessed with Pearson’s correlation coefficient. Assessments included absolute change in median lyso-Gb1 values, haemoglobin concentration, platelet count, and ferritin and chitotriosidase levels (from the first to last lyso-Gb1 assessment). Fold-change in median lyso-Gb1 values was calculated as follows: last value—baseline value/baseline value. All statistical analyses were conducted using NCSS software (v21.0.2; NCSS, LCC. Kaysville, UT, USA. ncss.com/software/ncss); the null hypothesis was rejected when the *p*-value was ≤0.05.

## 3. Results

### 3.1. Study Participants’ Characteristics

One hundred and eleven patients were screened. Of these, 100 patients (54M:46F; 93GD1 and 7GD3) had at least one lyso-Gb1 measurement and were included in the analysis. With regard to GBA1 genotype, N370S/other was the most common across patients (36/100 [36%]), followed by N370S/N370S (23/100 [23%]), N370S/L444P (17/100 [17%]), L444P/L444P (6/100 [6%]), L444P/other (6/100 [6%]), and other genotypes (7/100 [7%]). The genotype was not available for five (5%) patients for whom the diagnosis of GD was confirmed on enzymology.

At the time of lyso-Gb1 measurement, 93 patients (50M:43F; median age at first lyso-Gb1 measurement, 44.8 years [IQR, 33.3 to 56.3]) were receiving a DMT (median treatment duration, 10.4 years [IQR, 5.7 to 21.1], while 7 patients (3M:4F; median age at first lyso-Gb1 measurement, 57.3 years [IQR, 44.2 to 61.2]) were naive. Notably, naive patients, all GD1, had been diagnosed at a later age compared with the treated patients, due to their milder form of GD that was mainly characterised by an enlarged spleen (6/7 [86%]) or mild thrombocytopenia alone at diagnosis (1/7 [14%]). Of the 93 patients receiving treatment at the time of the analysis, 68 (73%) were on ERT (45 on velaglucerase and 23 on imiglucerase), while 25 (27%) were on SRT (i.e., eliglustat). Of the 68 patients receiving ERT, 16 (24%) were receiving a dose <30 U/kg, 46 (68%) were receiving a dose between 30 and 60 U/kg, and 6 (<1%) were on a dose >60 U/kg. Those on a higher dose of ERT were patients with GD3 with severe systemic involvement (i.e., lung involvement, spine abnormalities, osteopenia/osteoporosis complicated by fragility fractures, and osteonecrosis) and were transitioning from UK paediatric centres where high-dose ERTs are used.

As expected, the naive group showed a lower platelet count (median platelet count 157 × 109/L [IQR, 71 to 183]) than the treated one (median platelet count 200.5 × 109/L [IQR, 163.5 to 238.8], *p* = 0.017) and higher ferritin and chitotriosidase levels (median ferritin 539.5 mcg/L [IQR, 216.5 to 890.5]; they showed a median chitotriosidase 3042 nmol/hr/mL (IQR, 1735.5 to 6803.5) compared with the treated group (median ferritin 176 mcg/L [IQR, 88 to 313], *p* = 0.049); and they showed a median chitotriosidase 680 nmol/hr/mL (IQR, 272.5 to 1192.5, *p* = 0.001). Lower haemoglobin levels were observed in the naive group (median haemoglobin 129 g/L [IQR, 117 to 146] compared with the treated group (median haemoglobin 139 g/L [IQR, 126 to 151.8], *p* = 0413, which was not statistically significant.

Study cohort characteristics are shown in Table 1.

### 3.2. Cross-Sectional Analysis of Lyso-Gb1

As expected, median lyso-Gb1 values were significantly higher in the naive group (median 195 ng/mL, IQR 48.6 to 388) compared with the treated group (median 47.1 ng/mL, IQR 23.1 to 89.7), *p* = 0.015.

In the treated group, a further subgroup analysis was conducted by sex, GD type, genotype, DMT type, ERT dose, duration of treatment, and spleen status. Although not statistically significant, lyso-Gb1 values were higher in females (median 53 ng/mL, IQR 21 to 99) than in males (median 40.2 ng/mL, IQR 23.2 to 69.8); and also higher in GD3 (median 83.4 ng/mL, IQR 36.7 to 92.9) compared with type 1 (median 46.1 ng/mL, IQR 22.6 to 83.2). The higher lyso-Gb1 values in females could have been an ascertainment bias, as the seven females with the highest lyso-Gb1 at baseline were all GD3 patients. Patients with homozygous N370S genotype had statistically significantly lower values of lyso-Gb1 (median 20.6, IQR 15.4 to 45) compared to those with N370S/other variants (median 52.4 ng/mL, IQR 29.6 to 128), *p* = 0.019. With regard to DMT, similar lyso-Gb1 concentrations were noted between patients receiving ERT (median 46.1 ng/mL, IQR 23.1 to 96.6) and SRT (median 47.8 ng/mL, IQR 21.7 to 74.8). The analysis by ERT dose showed non-statistically significantly higher lyso-Gb1 values in those receiving an ERT dose >60 U/kg (median 87.3 ng/mL, IQR 19.7 to 126) compared with those receiving <30 U/kg (median 34 ng/mL, IQR 24.4 to 58.6) and 30–60 U/kg (median 50.2, IQR 23.1 to 100.5). Significantly higher lyso-Gb1 values were noted in those being treated for 15 years or more (median 62.9 ng/mL, IQR 36.6 to 103) than in patients on DMTs for less than 15 years (median 35.1 ng/mL, IQR 20.3 to 73.9), *p* = 0.006. Finally, significantly higher lyso-Gb1 levels were observed in splenectomised patients (median 83.4 ng/mL, IQR 34.7 to 224.5) compared with those with an intact spleen (40.7 ng/mL, IQR 21.4 to 77.1), *p* = 0.044.

In the sub-cohort of GD1-treated patients, lyso-Gb1 values were similar in patients on ERT (median 45 ng/mL, IQR 22.4 to 98.4) and SRT (median 47.8 ng/mL, IQR 21.7 to 74.8); the values were greater in those receiving an ERT dose of 30–60 U/kg (52.4, IQR 23.3 to 100) than a dose of <30 U/kg (34, IQR 21.2 to 66.7), and in patients on treatment for ≥ 15 years (62.9, IQR 35.9 to 107) than <15 years (34.5, IQR 20.3 to 58.2).

**Figure 1:**
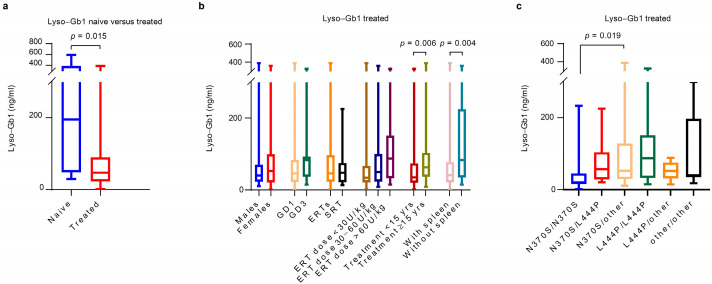
Cross-sectional lyso-Gb1 by sex, Gaucher disease type, DMT type, ERT dose, and duration of treatment at baseline. (**a**) The naive group showed significantly higher lyso-Gb1 values (median 195 ng/mL, IQR 48.6 to 388) compared with the treated one (median 47.1 ng/mL, IQR 23.1 to 89.7), *p* = 0.015. (**b**) In the treated group, significantly higher lyso-Gb1 values were observed in patients treated for more than 15 years (median 62.9 ng/mL, IQR 36.6 to 103) than in patients on DMTs for less than 15 years (median 35.1 ng/mL, IQR 20.3 to 73.9), *p* = 0.006. Higher lyso-Gb1 levels were also observed in splenectomised patients (median 83.446.1 ng/mL, IQR 34.7 to 224.5) compared with those with an intact spleen (40.746.1 ng/mL, IQR 21.4 to 77.1), *p* = 0.044. (**c**) In the treated group, patients with N370S/N370S genotype had significantly lower levels of lyso-Gb1 (median 20.6 ng/mL, IQR, 15.4 to 45) compared with those with N370S/other genotypes (median 52.4 ng/mL (29.6 to 128), *p* = 0.019. DMT: disease modifying therapy; ERT: enzyme replacement therapy; GD: Gaucher disease; SRT: substrate reduction therapy.

See Table 2 and Figure 1 for details.

### 3.3. Correlation with Other GD Biomarkers

**Figure 2:**
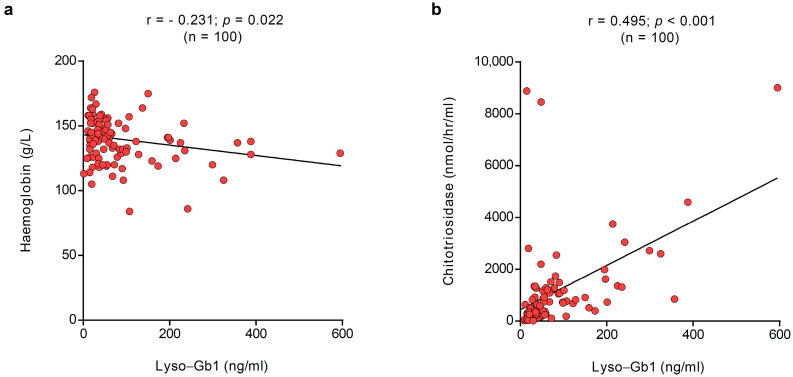
Correlation between plasma and dried blood spot lyso-Gb1. In the overall population, lyso-Gb1 correlated at baseline with (**a**) haemoglobin levels (r = −0.231; *p* = 0.022) and (**b**) chitotriosidase (r = 0.495; *p* < 0.001). The red dots represent the specific values for the variables for each subject; the lines represent the overall pattern in the relationship between variables.

All patients had contemporaneous biochemistry and lyso-Gb1 at baseline. Lyso-Gb1 correlated with haemoglobin levels (r = −0.231; *p* = 0.022) and chitotriosidase (r = 0.495; *p* < 0.001), as shown in Figure 2. No correlations with lyso-Gb1 were noted with platelet count (r = −0.1490; *p* = 0.141) and ferritin (r = 0.137; *p* = 0.190).

### 3.4. Changes in Lyso-Gb1 from Baseline to Visit

**Figure 3:**
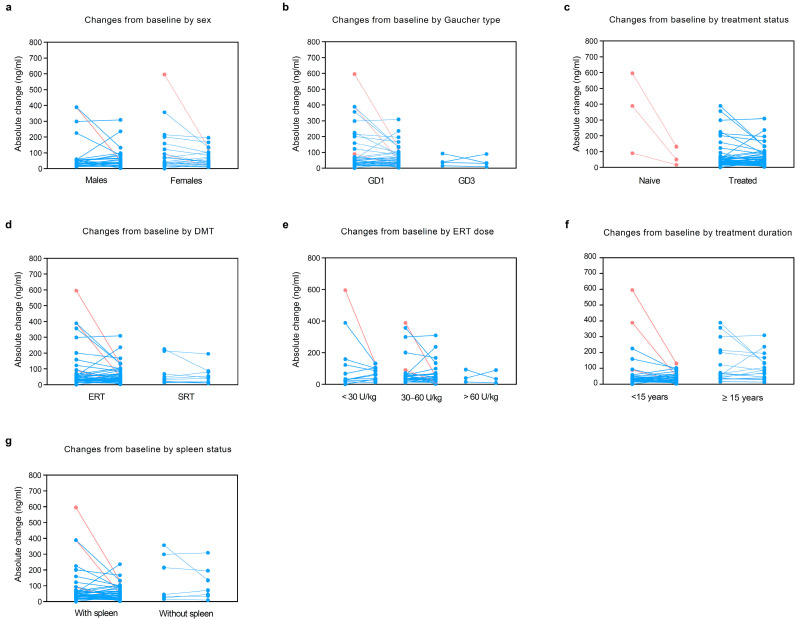
Changes in lyso-Gb1 from baseline to visit by sex, Gaucher disease type, treatment status, DMT type, ERT dose, duration of treatment, and spleen status. In a subset of 50 patients with paired data, we evaluated changes in lyso-Gb1 levels from baseline to visit by (**a**) sex, (**b**) Gaucher disease type, (**c**) treatment status, (**d**) DMT type, (**e**) ERT dose, (**f**) treatment duration, and (**g**) spleen status. Light red: naive patients; light blue: treated patients. DMT: disease-modifying therapies; ERT: enzyme replacement therapy; GD: Gaucher disease; SRT: substrate reduction therapy.

In a subset of 50 patients (3 naive and 47 treated) with paired values, changes in lyso-Gb1 values from baseline to visit were evaluated. In the overall subset, lyso-Gb1 decreased from baseline (median absolute change in lyso-Gb1 −1.7, IQR −24.5 to 14.8; median fold-change in lyso-Gb1 −0.1, IQR −0.4 to 0.5) over a median period of time of 2.9 years (IQR 1 to 4.2). The analysis by sex revealed a decrease in lyso-Gb1 in females (median absolute change −5.2, IQR −35.2 to 11.2), while a slight increase was noted in males (median absolute change 2.9, IQR −10.6 to 23.1). GD3 patients showed a greater decrease in lyso-Gb1 (median absolute change −5.8, IQR −45.9 to 37) compared with type 1 (median absolute change −0.3, IQR −24.5 to 14.8). Patients with homozygous L444P and N370S/L444P showed a greater reduction in lyso-Gb1 values (respectively, median absolute change −6.1 [IQR, −59.2 to 51.1] and median absolute change −9.7 [IQR, −32.1 to 16.2]) compared to other variants. As expected, a statistically greater reduction in lyso-Gb1 was observed in naive patients (median absolute change −338.6, IQR −464 to −73.5; median fold-change −0.8, IQR −0.9 to −0.8) compared with treated patients over time (median absolute change 0.4, IQR −13.3 to 15; median fold-change 0, IQR −0.3 to 0.5), *p* = 0.006. Interestingly, patients receiving SRT showed a decrease in lyso-Gb1 (median absolute change −3.5, IQR −19 to 8.1, *p* = 0.374), while a non-significant increase was observed in those receiving ERT (median absolute change 1, IQR −34 to 22.3, *p* = 0.625). Patients receiving a higher dose of ERT (>60 U/kg), i.e., GD3, showed a slightly higher decrease in lyso-Gb1 (median absolute change −6.1, IQR −59.2 to 51.1) compared with those receiving <30 U/kg (median absolute change 1.4, IQR −58 to 32.2) and 30–60 U/kg (median absolute change 1, IQR −20.5 to 13.2). Notably, splenectomised patients showed an increase in lyso-Gb1 (median absolute change 1.4, IQR, −19 to 22.3) compared with those with an intact spleen (median absolute change −2.5, IQR, −27.8 to 14.7). Haemoglobin levels, platelet count, and chitotriosidase levels showed improvements comparable to those observed for lyso-Gb1. See Table 3 and Figure 3 for details.

## 4. Discussion

In this study, we showed the utility of lyso-Gb1 as a biomarker of GD. Our results suggested a correlation with disease burden, as we observed a relationship with GD type, genotype, spleen status, treatment status, dose, duration of treatment, and baseline chitotriosidase level. Specifically, higher levels of lyso-Gb1 were observed in GD3, naive patients, those who had been splenectomised, those treated with higher doses of ERT (>60 U/kg) and for ≥15 years, and those with higher chitotriosidase levels. Interestingly, the greater levels of lyso-Gb1 observed in those treated with higher doses of ERT (>60 U/kg) included GD3 patients with severe systemic disease (skeletal abnormalities and haematological abnormalities) who had been transitioned from paediatric centres where high-dose ERTs (up to 120 U/kg every other week) are used. When they are transferred to adult centres, unless there are specific clinical needs, GD3 patients are usually allowed to grow into the standard 60 U/kg EOW dose, instead of a dose reduction: this result further confirms that lyso-Gb1 levels are driven by disease severity since a personalised approach to dosing has been practised for more than 25 years in the UK, with only selected GD patients (e.g., those with severe disease or showing an inadequate response to therapy) receiving higher doses of ERT [28,29].

These findings concur with previous evidence, with lyso-Gb1 levels being higher in patients with neuronopathic compared with non-neuronopathic disease, correlating with severe phenotype and treatment status, and, therefore, are useful for guiding treatment-related decisions [24,25,30,31].

As a further confirmation of the link between lyso-Gb1 and disease severity, we found that patients with homozygotes for N370S, who generally present a milder form of the disease [32], showed lower lyso-Gb1 levels than compound heterozygotes for the N370S variant, which is consistent with a recent analysis of lyso-Gb1 data from the Gaucher Outcome Study, an international disease-specific registry for GD patients [25].

At baseline, defined as the first lyso-Gb1 measurement, correlations were also noted between lyso-Gb1 concentrations and haemoglobin and chitotriosidase levels.

Lyso-Gb1 could also be useful for monitoring responses to treatment, and we observed a decrease in lyso-Gb1 over time in patients on DMTs. This was more evident in naive patients, where a 0.8-fold decrease from baseline was found, confirming that the most pronounced response is observed within the first months of initiating a DMT [24]. These results are consistent with previous published reports that found that naive patients display greater lyso-Gb1 values at baseline and a more pronounced reduction after treatment initiation compared with patients already receiving DMTs that may have experienced a more evident reduction in lyso-Gb1 before the first lyso-Gb1 assessment [27,31,33]. However, more data is needed in naive GD patients starting DMTs to provide guidance on the percentage decrease in lyso-Gb1 as a new therapeutic goal. Similar trends in biomarker response were observed for chitotriosidase, along with small improvements in haemoglobin concentrations, platelet counts, and ferritin levels. However, ferritin is a non-specific indicator of GD as it can also be elevated in other conditions, such as inflammation, while the accuracy of chitotriosidase, which is an enzyme produced and secreted by activated macrophages, is limited by the presence of genetic variants in the CHIT1 gene, such as the common 24 base-pair duplication often found in individuals of European and Asian descent, which results in a significant reduction in chitotriosidase activity in carriers compared with individuals without the mutation [21,22]. Nonetheless, as the chitotriosidase test is a cost-effective procedure that is widely available through major diagnostic laboratories and hospitals, chitotriosidase values should be investigated for diagnostic confirmation and for monitoring disease trajectory, especially when lyso-Gb1 analysis cannot be performed.

In our cohort, although lyso-Gb1 decreased over time, it remained significantly above the normal range, despite improved biochemistry and clinical stability. Moreover, the percentage reduction in lyso-Gb1 in GD1 patients on less than 30 units/kg and 30 to 60 units/kg was comparable, with no statistically significant difference. We speculate that the persistently increased lyso-Gb1 levels may have been driven by underlying mechanisms, such as chronic inflammation caused by substrate accumulation and subsequent macrophage activation, that are not completely reversed by DMTs [34].

Finally, we have also demonstrated that lyso-Gb1 in DBS offers easy sampling and shipping, along with sample stability, compared with other methods such as plasma or serum. DBS lyso-Gb1 can, therefore, be easily measured at clinical appointments as part of the diagnostic work-up, monitoring disease progression and responses to treatment.

This study has several limitations. Firstly, there was a small sample size of naive patients with available data for analysis compared with the over-represented group of treated patients. Lyso-Gb1 is measured infrequently during routine clinic appointments. This could have potentially impacted the ability to accurately evaluate changes in lyso-Gb1 over time. Also, a longer follow-up with more clinical parameters could have provided more insights into correlations between lyso-Gb1 values, a relatively new biomarker, and haematology, organomegaly, and bone disease, which have previously been included in therapeutic goals of DMTs in GD. In addition, the presence of anti-drug antibody (ADA)-neutralising ADA, which is known to be associated with an attenuation of the efficacy of ERTs and worse clinical outcomes, was not tested in this cohort. ADA measurement is not routinely offered by independent laboratories in the UK and often relies on the test being sent to the relevant ERT pharmaceutical companies. However, in our cohort, we observed no infusion-associated reactions in enzyme replacement therapy.

The strength of this study is that lyso-Gb1 was measured using the same method in a single laboratory, thus limiting the variability due to different methodologies employed by different laboratories.

## 5. Conclusions

Lyso-Gb1 concentrations are reflective of disease severity and treatment status, with naive patients showing greater lyso-Gb1 values at baseline and the most pronounced reduction after treatment initiation. Determining the reasons for non-normalisation of lyso-Gb1 values in the context of clinical stability requires further investigations, including detailed analyses of the inflammatory pathways affecting lysosomal function.

## Figures and Tables

**Table 1:** Patient demographics and characteristics at baseline.

Variable	Naïve n = 7	Treated n = 93	*p*-Value
Age at first lyso-Gb1 measurement (years), median (IQR)	57.3 (44.2 to 61.2)	44.8 (33.3 to 56.3)	0.129 #
Sex, N (%)			0.577 +
Male	3 (43%)	50 (54%)	
Female	4 (57%)	43 (46%)	
Gaucher disease type, n (%)			0.488 +
GD type 1	7 (100%)	86 (94%)	
GD type 3	0	7 (6%)	
Genotype, N (%)			0.494 +
N370S/N370S	3 (43%)	20 (22%)	
N370S/L444P	-	17 (18%)	
N370S/other	3 (100%)	33 (35.5%)	
L444P/L444P	-	6 (6.5%)	
L444P/other	-	6 (6.5%)	
other/other	-	7 (7.5%)	
not available	1 (14%) *	4 (4%) *	
Time to first lyso-Gb1 measurement (years), median (IQR)	−0.2 (−0.7 to 0)	-	-
Duration of treatment prior to first lyso-Gb1 measurement (years), median (IQR)	-	10.4 (5.7 to 21.1)	-
Haemoglobin (g/L), median (IQR)	129 (117 to 146)	139 (126 to 151.8)	0.413 #
Platelet count (×10^9^/L), median (IQR)	157 (71 to 183)	200.5 (163.5 to 238.8)	0.017 #
Ferritin (µg/L), median (IQR)	539.5 (216.5 to 890.5)	176 (88 to 313)	0.049 #
Chitotriosidase (nmol/hr/mL), median (IQR)	3042 (1735.5 to 6803.5)	680 (272.5 to 1192.5)	0.001 #
Lyso-Gb1 (ng/mL), median (IQR)	195 (48.6 to 388)	47.1 (23.1 to 89.7)	0.015 #
Splenectomy, N (%)			0.289 +
No	7 (100%)	80 (86%)	
Yes	0	13 (14%)	

Continuous variables presented as median (IQR, interquartile range). Categorical variables are presented as a number (%). + Chi-square test; # Mann–Whitney test. * Patients with missing genotypes were excluded from the analysis. GD: Gaucher disease.

**Table 2:** Cross-sectional lyso-Gb1 values (ng/mL) by treatment status and by sex, Gaucher disease type, DMTs, ERT dose, duration of treatment, and spleen status at baseline.

Variable	Lyso-Gb1 (ng/mL), Median (IQR)	*p*-Value	Lyso-Gb1 (ng/mL), Median (IQR)	*p*-Value
	Naive		Treated	
Sex		1.000		0.606 #
Male	241.7 (29.5 to 388); n = 3		40.2 (23.2 to 69.8); n = 50	
Female	142.65 (59.03 to 495); n = 4		53 (21 to 99); n = 43	
Gaucher disease type		-		0.348 #
GD type 1	195 (48.6 to 388); n = 7		46.1 (22.6 to 83.2); n = 86	
GD type 3	-		83.4 (36.7 to 92.9); n = 7	
Genotype		0.700 #		0.019 ‡
N370S/N370S	90.3 (48.6–195); n = 3		20.6 (15.4 to 45) ‡; n = 20	
N370S/L444P	-		56.8 (28.6 to 104); n = 17	
N370S/other	241.7 (29.5 to 595); n = 3		52.4 (29.6 to 128) ‡; n = 33	
L444P/L444P	-		87.3 (32.7 to 150.9); n = 6	
L444P/other	-		52.2 (31.2 to 75.1); n = 6	
other/other	-		39.5 (34.5 to 197); n = 7	
not available	388 (388 to 388) *; n = 1		68.4 (31.6 to 116.4) *; n = 4	
DMTs		-		0.812 #
ERTs	-		46.1 (23.1 to 96.6); n = 68	
SRT	-		47.8 (21.7 to 74.8); n = 25	
ERT dose		-		0.326 ‡
<30 U/kg	-		34 (24.4 to 58.6); n = 16	
30–60 U/kg	-		50.2 (23.1 to 100.5); n = 46	
>60 U/kg	-		87.3 (19.7 to 126); n = 6	
Duration of treatment		-		0.006 #
<15 years	-		35.1 (20.3 to 73.9); n = 56	
≥15 years	-		62.9 (36.6 to 103); n = 37	
Splenectomy		-		0.044 #
No	195 (48.6 to 388); n = 7		40.7 (21.4 to 77.1); n = 80	
Yes	-		83.4 (34.7 to 224.5); n = 13	

Continuous variables presented as median (IQR, interquartile range). # Mann–Whitney test; ‡ Kruskal–Wallis one-way ANOVA test. * Patients with missing genotypes were excluded from the analysis. DMT: disease-modifying therapy; ERT: enzyme replacement therapy; GD: Gaucher disease; SRT: substrate reduction therapy.

**Table 3:** Absolute changes in lyso-Gb1 from baseline to visit by sex, Gaucher disease type, treatment status, DMTs, ERT dose, duration of treatment, and spleen status.

Variable	Lyso-Gb1 (ng/mL), Median (IQR)	Haemoglobin (g/L), Median (IQR)	Platelet Count (×10^9^/L), Median (IQR)	Ferritin (mcg/L), Median (IQR)	Chitotriosidase (nmol/hr/mL), Median (IQR)
Overall n = 50	−1.7 (−24.5 to 14.8)	2.5 (−3.3 to 8)	5 (−9.3 to 24)	−4 (−116.5 to 45.5)	−236 (−1337 to 187)
Sex					
Male (n = 25)	2.9 (−10.5 to 23.1)	1 (−3.5 to 6.5)	3 (−11.5 to 18.5)	−4 (−154 to 48)	−61.5 (−1368 to 206.5)
Female (n = 25)	−5.2 (−35.2 to 11.2)	4 (−4 to 8.5)	7 (−8 to 31.5)	−7.5 (−77.8 to 42.5)	−730 (−1184 to 134.5)
Gaucher disease type					
GD type 1 (n = 46)	−0.3 (−24.5 to 14.8)	2 (−4 to 8.3)	5 (−9.3 to 23)	−4 (−116.5 to 48)	−341 (−1400 to 172.3)
GD type 3 (n = 4)	−5.8 (−45.9 to 37)	−5.8 (−45.9 to 37)	3 (1.3 to 6.3)	17.5 (−27 to 50)	−7.5 (−151.8 to 34)
Genotype					
N370S/N370S (n = 12)	−3 (−9.8 to 26.4)	−0.5 (−10.8 to 3)	−2.5 (−33 to 16)	0 (−151 to 46)	−299 (−8463 to 0)
N370S/L444P (n = 9)	−9.7 (−32.1 to 16.2)	1 (−4.5 to 6)	6 (−7.5 to 29.5)	−29 (−100.5 to 115)	36 (−360.5 to 311)
N370S/other (n = 19)	1 (−34 to 11.3)	4 (−3 to 8)	7.5 (0.8 to 23)	−4 (−56 to 85)	−819 (−1368 to 38.5)
L444P/L444P (n = 3)	−6.1 (−59.2 to 51.1)	2 (1 to 7)	7 (1 to 23)	−16 (−197 to 45)	3 (−133 to 873)
L444P/other (n = 3)	7.6 (−5.5 to 25.7)	9 (4 to 10)	−37 (−79 to 19)	−43 (−78 to 1)	586 (586 to 586)
other/other (n = 3)	10 (−7.3 to 14.7)	11 (4 to 31)	1 (−10 to 290)	25 (15 to 181)	208 (−1709 to 266)
missing (n = 1)	−338.6 (−338.6 to −338.6)	−19 (−19 to −19)	51 (51 to 51)	−741 (−741 to −741)	−4098 (−4098 to −4098)
Treatment status					
Naive (n = 3)	−338.6 (−464 to −73.5)	7 (−19 to 23)	19 (4 to 51)	−147 (−741 to −125)	−4098 (−9015 to −1421)
Treated (n = 47)	0.4 (−13.3 to 15)	2 (−3 to 8)	4 (−10 to 23)	0.5 (−77.25 to 47)	−100 (−891 to 202.8)
DMTs					
ERTs (n = 39)	1 (−34 to 22.3)	4 (−1 to 10)	4 (−13 to 28)	−1.5 (−122.8 to 61)	−133 (−947 to 265)
SRT (n = 11)	−3.5 (−19 to 8.1)	−1 (−6 to 1)	6 (0 to 23)	−48 (−74 to 15)	−516.5 (−1430 to 140.3)
ERT dose					
<30 U/kg (n = 11)	1.4 (−58 to 32.2)	4 (−5 to 7)	4 (−17 to 10)	−1.5 (−150 to 61)	−512.5 (−2964 to 422)
30–60 U/kg (n = 25)	1 (−20.5 to 13.2)	4 (−0.5 to 11)	3 (−13.5 to 27.5)	1 (−94.5 to 126.5)	−225 (−1493 to 265.3)
>60 U/kg (n = 3)	−6.1 (−59.2 to 51.1)	2 (1 to 7)	32 (−3 to 56)	−16 (−197 to 45)	0 (−133 to 873)
Duration of treatment					
<15 years (n = 31)	0.4 (−13.3 to 15)	1 (−3 to 7)	4 (−17 to 22)	−51.5 (−148 to 17.3)	−183 (−1451 to 202.8)
≥15 years (n = 19)	−3.5 (−27.8 to 10.1)	4 (−4 to 9)	6 (0 to 28)	25 (−35 to 180)	−236 (−947 to 128)
Splenectomy					
No	−2.5 (−27.8 to 14.7)	2 (−4 to 7)	4 (−10 to 19)	−2 (−124.3 to 42.3)	−133 (−947 to 187)
Yes	1.4 (−19 to 22.3)	4 (1 to 9)	31 (19 to 173)	−16 (−77 to 248)	−1279 (−1719.5 to 442.5)

Continuous variables presented as median IQR (interquartile range). DMT: disease-modifying therapies; ERTs: enzyme replacement therapy; SRT: substrate reduction therapy.

## Data Availability

Data will be made available by the senior author on reasonable.
